# A Phase I Comparative Pharmacokinetic and Safety Study of Two Intravenous Formulations of Vinorelbine in Patients With Advanced Non-Small Cell Lung Cancer

**DOI:** 10.3389/fphar.2019.00774

**Published:** 2019-07-11

**Authors:** Guolan Wu, Lihua Wu, Huili Zhou, Meihua Lin, Ling Peng, Yina Wang, You Zhai, Xingjiang Hu, Yunliang Zheng, Duo Lv, Jian Liu, Jianzhong Shentu

**Affiliations:** ^1^Research Center of Clinical Pharmacy, State Key Laboratory for Diagnosis and Treatment of Infectious Disease, First Affiliated Hospital, College of Medicine, Zhejiang University, Hangzhou, China; ^2^Zhejiang Provincial Key Laboratory for Drug Evaluation and Clinical Research, First Affiliated Hospital, College of Medicine, Zhejiang University, Hangzhou, China; ^3^Department of Radiation Oncology, First Affiliated Hospital, College of Medicine, Zhejiang University, Hangzhou, China; ^4^Department of Oncology, First Affiliated Hospital, College of Medicine, Zhejiang University, Hangzhou, China

**Keywords:** pharmacokinetics, bioavailability, safety, vinorelbine, non-small-cell lung cancer

## Abstract

**Purpose:** The aim of this study was to compare the pharmacokinetics and safety between two vinorelbine formulations [a new oil-in-water emulsion formulation (ANX) versus a previously marketed solution formulation (Navelbine)] in Chinese patients with advanced non-small cell lung cancer (NSCLC).

**Method:** This was a single-center, randomized, open-label study. Eligible patients aged 18–70 years who had histologically or cytologically confirmed NSCLC were enrolled. In cycle 1, the patients alternatively received the two formulations (30 mg/m^2^, given as a 10-min infusion) with a 7-day interval. Samples for pharmacokinetic analysis were taken during cycle 1. For all subsequent 21-day cycles (maximum four cycles), ANX was administered on days 1 and day 8. Bioequivalence analysis was performed on C_max_, AUC_last_, and AUC_inf_. The safety profiles and anti-tumor effects were also determined.

**Results:** From March 2013 to January 2015, 24 patients were enrolled and 20 were eligible for pharmacokinetic evaluation. The 20 subjects in the pharmacokinetic analysis set had a median age of 61 years (range, 37–70 years), and 15 patients were male (75%). Mean vinorelbine C_max_ values for ANX and Navelbine were 1,317.40 and 1,446.30 ng/mL, respectively. Corresponding AUC_last _values were 797.08 and 924.26 ng·h/mL, respectively. AUC_inf _values were 830.14 and 957.16 ng·h/mL, respectively. Treatment ratios of the geometric means were 90.00% (90% CI, 83.22–99.07%) for C_max_, 86.92% (90% CI, 80.91–93.37%) for AUC_last_, and 87.44% (90% CI, 82.08–93.16%) for AUC_inf_. These results met the required 80–125% bioequivalence criteria. The most frequently reported adverse events after vinorelbine administration were neutropenia, leucopenia, neutropenic fever, and constipation.

**Conclusion:** At therapeutic dosage levels, pharmacokinetic behavior and safety profiles were similar for both formulations. Chinese National Registry Code: ChiCTR-IPR-15005856.

## Introduction

Worldwide, lung cancer is the most commonly diagnosed cancer (11.6% of the total cases) and the leading cause of cancer death (18.4% of the total cancer deaths) (Bray et al., 2018). Nonsmall cell lung cancer (NSCLC) accounts for ∼85% of all lung cancers (Schvartsman et al., 2016). Nearly 60% of NSCLC cases are metastatic at diagnosis, and the 5-year relative survival rate for metastatic disease is 4.2% (Kaburaki et al., 2017). Many advances have been made in the first-line systemic treatment of NSCLC, and current treatment approaches are markedly different from what they were several years ago. At the onset of the 2000s, first-line management of patients with advanced NSCLC relied on platinum-based doublets chemotherapy. In the last decade, targeted therapies have transformed the management of patients whose tumors harbor an oncogenic mutation (∼15%), but the majority of patients cannot benefit from these treatments (Bylicki et al., 2019). For patients without driver oncogenes, however, improvements in survival were minimal until immunotherapeutic options became available. Since then, these treatments have quickly moved to the first-line setting (Borghaei et al., 2015; Brahmer et al., 2015; Fehrenbacher et al., 2016; Mok et al., 2019). Yet, it is only a minority of patients who achieve this transcendent, durable benefit from immunotherapy. Overall, the prognosis for lung cancer remains poor. Nevertheless, most lung cancer patients will still need, and continue to receive, chemotherapy over their disease course (Hardin et al., 2017).

Vinorelbine, a semisynthetic vinca alkaloid, is known to be an active agent in NSCLC treatment (Crawford, 1996; Jones and Burris, 1996). Like other vinca, it exhibits cell cycle-specific cytotoxic activity by binding to tubulin and preventing its assembly into microtubules, ultimately inhibiting mitosis and inducing apoptosis (Caffo et al., 2013). Either used alone or in combination, vinorelbine has demonstrated clinical benefits in different settings of lung cancer management (Gridelli, 2001; Reck et al., 2014; Lesueur et al., 2018). Vinorelbine for intravenous (i.v.) administration by label is typically given 25–30 mg/m^2^ on days 1 and 8 of a 21-day cycle. Its main dose-limiting toxicity is grade 3 leukocytopenia, which develops 8–10 days after each dose. Like other anticancer vinca alkaloids, vinorelbine is a moderate vesicant that is well documented to cause local venous toxicity such as injection site reactions and chemical phlebitis (Winton et al., 2005; Yoh et al., 2007; Li et al., 2013). Although local venous toxicity is not life threatening, it can be discomforting or painful; thus, it may negatively influence patients’ quality of life. The oral formulation provides a valuable option for treating cancer patients because of ease of administration, greater convenience for the patient, and reduced need for hospitalization. However, relatively higher cost and incidence of gastrointestinal toxicities may reduce patient compliance. Consequently, improved i.v. formulations have been sought for vinorelbine. To overcome the local venous toxicity of vinorelbine, drug delivery systems were developed, such as nanoparticles, liposomes, microspheres, and lipid emulsions (Yang et al., 2012; Li et al., 2013). A new bitartrate vinorelbine emulsion has recently been developed in China. The vinorelbine is encapsulated in the oil droplets, thus preventing direct contact of drug molecules with the venous endothelium tissue, and therefore reducing the venous toxicity.

Accurate and comprehensive understanding of safety and PK profiles of new formulation is needed for clinical development. Hence, we conducted a phase I study to compare pharmacokinetic parameters of this new bitartrate emulsion with a previously marketed solution in NSCLC patients. The efficacy and safety profile of this new bitartrate emulsion was also evaluated as an exploratory objective. The study will provide a basis for better clinical use of the drug in Chinese population in the future.

## Patients and Methods

The study protocol and its amendments were reviewed and approved by the ethics committee of the First Affiliated Hospital, School of Medicine, Zhejiang University (approval no.: 2012-EC-60). The trial was designed according to the current revised Declaration of Helsinki and conducted in accordance with Good Clinical Practice guidelines. Written informed consent was obtained from each participating patient before entry into the study.

### Patient Selection

The study was initiated in March 2013 and closed to accrual in February 2015. All enrolled patients had histologically or cytologically confirmed non-small cell lung cancer. Additional inclusion criteria included the following: age ≥18 and ≤70 years; a performance status ≤2 (ECOG-Zubrod scale); life expectancy greater than 3 months; initial blood parameters of hemoglobin ≥10 g/dl, granulocytes (absolute neutrophil count) ≥1.5 × 10^9^/L, platelets ≥80 × 10^9^/L; adequate hepatic function and renal function [bilirubin < 1.5 mg/dl, transaminases <2.5 times upper limit of normality (ULN) or ≤5.0 × ULN if liver metastases present]; serum total bilirubin ≤1.5 × ULN; calculated creatinine >80 ml/min (Cockcroft–Gault formula); and presence of at least one evaluable or measurable lesion according to Response Evaluation Criteria in Solid Tumors (RECIST) guidelines.

Additional ineligibility criteria included a history of primary central nervous system tumors or brain metastases, any peripheral neuropathy of grade ≥1 per the Common Terminology Criteria for Adverse Events (CTCAE), impaired cardiac function [left ventricular ejection fraction <45%, complete left bundle branch block, obligate use of a cardiac pacemaker, congenital long QT syndrome, history or presence of significant ventricular or atrial tachyarrhythmias, clinically significant resting bradycardia (<50 beats per minute), QTcF >480 ms on screening electrocardiogram, or other clinically significant heart disease], impairment of gastrointestinal function or gastrointestinal disease, and acute or chronic liver or renal disease. Female subjects with reproductive potential were required to have a negative pregnancy test (urine and/or serum β-human chorionic gonadotropin) and agree to use an effective (>90% reliability) form of birth control during the study. Subjects with an acute infection or fever over 38.0°C within 3 days prior to study entry, cardiovascular disease, lactation, or history of allergic reactions attributed to compounds of similar chemical or biologic composition to vinorelbine were not eligible for the study. Subjects with positive tests for human immunodeficiency virus, hepatitis B virus surface antigen, or anti-hepatitis C virus antibody were also excluded. Patients were required to be able to understand and sign a written informed consent document.

### Pretreatment and Follow-Up Evaluation

Pretreatment evaluation included a complete medical history and physical examination, a computed tomographic (CT) scan of the chest and abdomen, magnetic resonance imaging (MRI) of the brain, and a bone scintigraphy to assess the extent of disease. Follow-up consisted of physical examination, monitoring of toxic effects, a complete blood count, liver function tests, chest radiography, and CT scan as clinically indicated. Tumor response was assessed using RECIST criteria. Tumor responses were assessed every two cycles of chemotherapy, and patients were evaluated before each new treatment cycle for toxicities.

### Study Drug Administration/Treatment Plan

Vinorelbine treatment by label is typically given on days 1 and 8 of a 21-day cycle. In cycle 1, patients were randomized to receive a single i.v. dose of either vinorelbine bitartrate emulsion injection or vinorelbine solution, then 1 week later crossover to the alternate vinorelbine formulation. All drug administrations were given as 10-min infusions at the dose of 30 mg/m^2^. No dose reduction was allowed in the first cycle. In following cycles, patients were planned to receive vinorelbine bitartrate emulsion injection on days 1 and 8. Cycles were repeated every 3 weeks if the patient’s blood count had returned to normal and non-hematologic toxicities had resolved. Dosage of subsequent cycles was adjusted according to the observed toxicities that developed during the preceding cycle. The criteria for dose reduction included grade 4 hematological and grade 3 non-hematological toxicity, in which case the vinorelbine dose was reduced by 20%.The treatment continued for a maximum of four cycles or until disease progression.

### Pharmacokinetics

Vinorelbine pharmacokinetic (PK) parameters were evaluated based on collection of plasma samples during cycle 1. Blood samples (3 ml) were collected from an indwelling venous catheter into heparinized tubes immediately before (within 15 min) the start of infusion and 5, 10, and 30 min, and 1, 2, 3, 6, 12, 24, 48, 72, 96, 120, and 144 h after the start of the infusion for each treatment on days 1 and 8. Vinorelbine concentration was determined using a validated liquid chromatography/mass spectrometry-mass spectrometry (LC/MS-MS) analytical procedure. The assay was validated in the range of 0.36–1,450.38 ng/ml. Pharmacokinetic parameters were calculated by non-compartment method using WinNonlin 6.3 software (Pharsight Corporation; Sunnyvale, CA, USA).

The primary outcome measure was the evaluation of the bioavailability of the test product and the reference product, assessed as extent of exposure (AUC_last_, area under the curve from time zero to last measurable concentration). Secondary pharmacokinetic outcomes were the pharmacokinetic parameters: maximum plasma concentration (C_max_), area under the curve from first time point extrapolated to infinity (AUC_inf_), plasma peak concentration time (T_max_), total body clearance (CL), apparent volume of distribution (V_z_), and terminal half-life (T_1/2_).

### Safety and Tolerability Variables

Injection-site tolerability was assessed in relation to six specific reactions: erythema,itching,swelling, bruising, bleeding, and pain. Pain was assessed using a visual analogue scale (VAS), where 0 mm was equal to no pain and 100 mm was equal to severe pain. Information on adverse events (AEs) was collected throughout the study. Additional safety variables included vital signs, physical examination, 12-lead electrocardiogram, hematology, clinical chemistry, urinalysis, and any general signs or symptoms relating to potential adverse effects of the drug.

### Statistical Analysis

All participants for whom primary PK parameters could be calculated for both treatment periods were included in the PK population. The safety population comprised all individuals who received at least one dose of study treatment. AEs were summarized by treatment. Bioequivalence was assessed according to the China Food and Drug Administration (CFDA) Guideline on the Investigation of Bioequivalence. Thus, 90% confidence intervals (CIs) for analysis of variance estimates of the geometric mean ratios of the primary PK parameters for the two products (test/reference) had to lie within the range of 80.00–125.00%. The geometric mean ratios were calculated from least square mean values. The required calculations were performed using Phoenix WinNonlin 6.3. Assuming the above bioequivalence criteria for C_max_ and AUC_last_, a within-participant coefficient of variation (CV%) of 20%, and a “test/reference” mean ratio between 0.95 and 1.05, it was calculated that 24 participants would be needed to show bioequivalence with a power of at least 80% and an alpha level of 0.05.

## Results

### Study Participants

**Figure 1:**
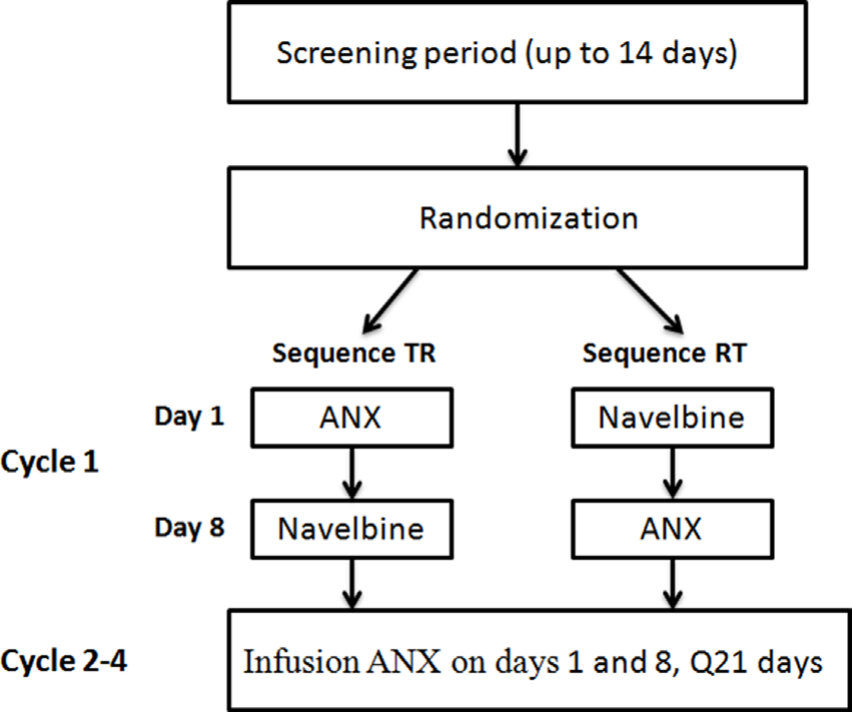
Study design.

Thirty individuals were screened for inclusion in the study, and 24 of them were randomized to the study (**Figure 1**). Six subjects were not randomized, the reasons being consent withdrawal (*n* = 2) and screening failures (*n* = 4). Four volunteers withdrew from the study before the second treatment in cycle 1. Therefore, 20 individuals completed the study and were included in the PK population. The baseline characteristics of the patients are summarized in **Table 1**. Nineteen patients were male and five were female, with a median age of 61 years (range, 37–70 years). The median body weight was 61.0 kg (range, 40.5–77.0 kg), and the median body surface area was 1.68 m^2^ (range, 1.34–1.82 m^2^).

**Table 1:** Baseline patient characteristics (*n* = 24).

Characteristics		No. of patients
Sex	Male/female	19/5
Age, years	Median (range)	61 (37–70)
ECOG PS	0/1	4/20
Histologic type	Adenocarcinoma	24
Clinical stage	III/IV	7/17
Height, cm	Median (range)	167.5 (146–173)
Weight, kg	Median (range)	61.0 (40.5–77.0)
BSA, m^2^	Median (range)	1.68 (1.34–1.82)
Prior chemotherapy	Yes/no	22/2
Prior radiotherapy	Yes/no	11/13
Prior surgery	Yes/no	14/10
Duration of treatment (cycles)	0/1/2/3/4	4/4/2/4/10

ECOG PS, Eastern Cooperative Oncology Group performance status; BSA, body surface area.

Duration of treatment was defined as the total cycles that the study drug was taken.

### Dose Administration

The total number of cycles delivered to all patients was 60, with a median number of three treatment cycles delivered per patient (range, 0–4). After the first administration of 30 mg/m^2^ vinorelbine, four patients (three patients using Navelbine and one patient using ANX) were withdrawn from the study due to either safety reasons or patient decision. As per protocol, a total of 11 patients (45.8%) had a vinorelbine dose reduction. Seven patients had dose reduction for the second cycle due to grade 3 neutropenia (two patients) and grade 4 neutropenia (five patients).One additional dose reduction was applied for cycle 3 in one patient for whom vinorelbine was administered at 25 mg/m^2^ at cycle 2 due to grade 4 neutropenia. **Table 2** displays an overview of dose changes during the study in patients.

**Table 2:** Evolution of vinorelbine dose schedule during the study in patients.

Evolution of vinorelbine dose schedule during study starting dose at cycle 1 = 30 mg/m^2^	*n*	%
Decrease to 25 mg/m^2^ at cycle 2 up to cycle 3	4	16.7
Decrease to 25 mg/m^2^ at cycle 2 up to cycle 4	3	12.5
Decrease to 25 mg/m^2^ at cycle 2 day 1, decrease to 18.75 mg/m^2^ at cycle 2 day 8	1	4.2
Decrease to 25 mg/m^2^ at cycle 2, decrease to 20 mg/m^2^ at cycle 3 up to cycle 4	1	4.2
No change up to cycle 2	1	4.2
No change up to cycle 2, decrease to 25 mg/m^2^ at cycle 3 up to cycle 4	1	4.2
No change up to cycle 3 day 1, decrease to 25 mg/m^2^ at cycle 3 day 8	1	4.2
No change up to cycle 4	4	16.7
Not treated after cycle 1	4	16.7
Not treated after cycle 1 day 1	4	16.7
Number of patients	24	100.0

### Pharmacokinetic Analysis

**Figure 2:**
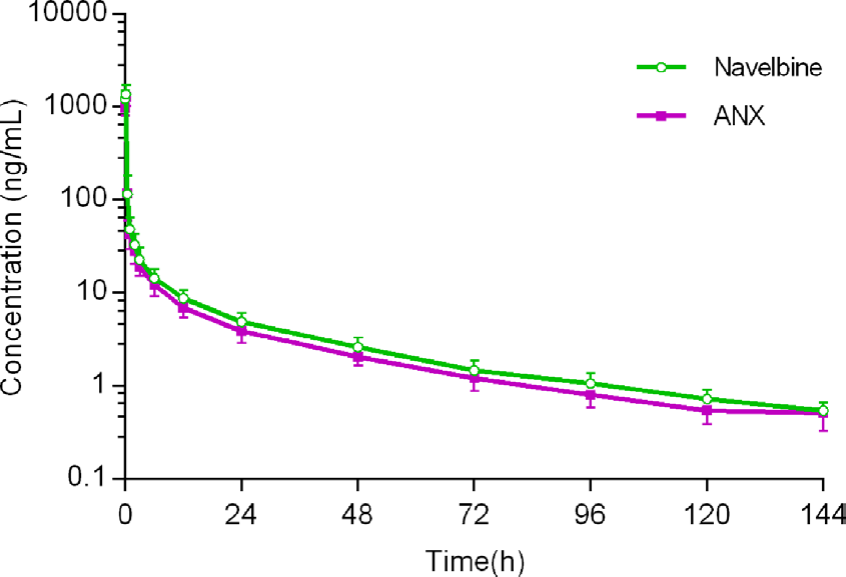
Mean plasma vinorelbine concentration vs time curves after receiving ANX and Navelbine.

**Figure 3:**
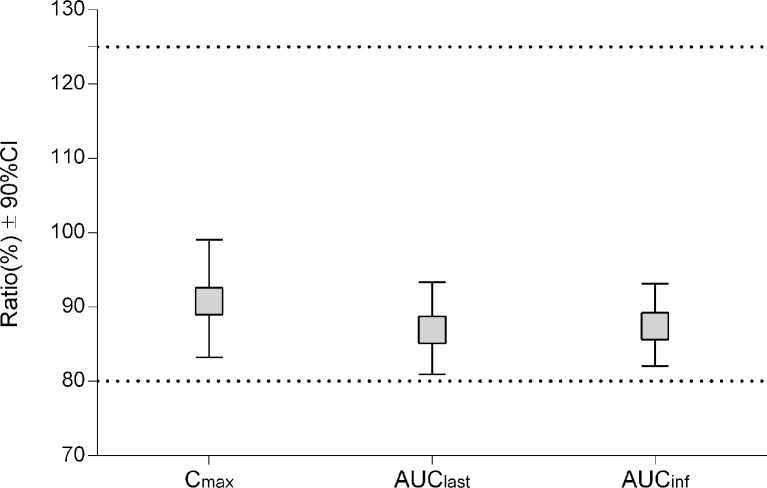
Pharmacokinetic equivalence between ANX and Navelbine.

**Figure 4:**
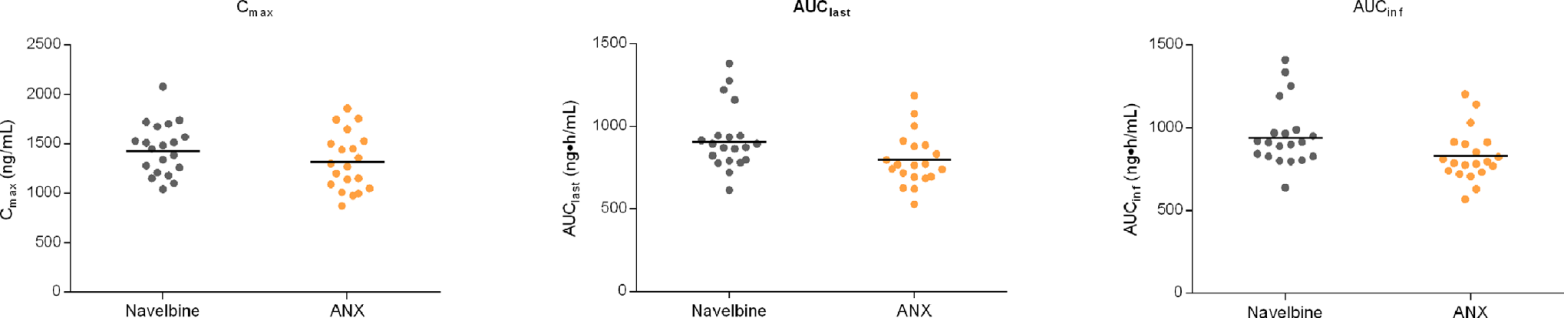
Individual pharmacokinetic parameters.

For the first cycle, 20 patients provided full and interpretable PK results. The mean plasma concentration–time curves of vinorelbine after an i.v. administration of 30 mg/m^2^ of the two formulations to Chinese NSCLC patients are shown in **Figure 2**. The main PK parameters calculated for both formulations are listed in **Table 3**. Similar T_max_ results were observed for the two formulations. The 90% CIs of the test/reference (ANX versus Navelbine) ratios of the natural log-transformed values of AUC_last_, AUC_inf_, and C_max_ were within the predetermined bioequivalence range of 80.00–125.00% (**Figure 3** ****and**** **Table 3**). Individual values of vinorelbine pharmacokinetic parameters (AUC_last_, AUC_inf_, and C_max_) obtained in cycle 1 are depicted in **Figure 4**. Therefore, it was deduced that the test and reference formulations were bioequivalent according to the guidelines.

**Table 3:** Pharmacokinetic results for the two vinorelbine products.

Parameter	Navelbine	ANX	Geometric mean ratio (%)	90% CI	Acceptable
C_max_ (ng/mL)	1,446.30 ± 260.85	1,317.40 ± 290.64	90.80	80.91–93.37	80–125
AUC_last_ (ng·h/mL)	924.26 ± 193.52	797.08 ± 159.12	86.92	82.08–93.16	80–125
AUC_inf_ (ng·h/mL)	957.16 ± 639.67	830.14 ± 156.25	87.44	83.22–99.07	80–125
T_max _(h)^a^	0.17 (0.08–0.17)	0.17 (0.08–0.17)			
V_z_ (L/kg)	0.89 ± 0.15	1.03 ± 0.21			
CL (L/h/kg)	51.43 ± 21.32	68.43 ± 54.43			
T_1/2_ (h)	40.17 ± 13.87	44.95 ± 32.51			

a T_max_ value = median (range).

### Safety and Tolerability

All enrolled patients were evaluable for safety. No toxic death occurred during the study, and no unexpected adverse events were recorded. Overall toxicity data are summarized in**** **Table 4**.

**Table 4:** Adverse events in all cycles of treatment for all patients receiving at least one treatment.

Adverse event	In cycle 1		In cycle 1		In following cycles	
	Navelbine (*N* = 23)		ANX (*N* = 21)		ANX (*N* = 16)	
	All grades, *n* (%)	Grades 3/4, *n* (%)	All grades, *n* (%)	Grades 3/4, *n* (%)	All grades, *n* (%)	Grades 3/4, *n* (%)
TEAEs	19 (82.6)	11 (47.8)	17 (80.9)	11 (52.4)	9 (56.3)	4 (25.0)
TEAEs leading to withdrawal	3 (13.0)	2 (8.7)	2 (9.5)	1 (4.8)	1 (6.25)	1 (6.25)
By organ system						
Administration site conditions						
Phlebitis	2 (8.7)	1 (4.3)	0 (0)	0 (0)	0 (0)	0 (0)
Blood and lymphatic system disorders						
Anemia	1 (4.3)	0 (0)	2 (9.5)	1 (4.2)	1 (6.3)	0 (0)
Leucopenia	14 (60.9)	8 (34.8)	12 (57.1)	9 (42.9)	7 (43.8)	4 (25.0)
Neutropenia	17 (73.9)	9 (39.1)	13 (61.9)	8 (38.1)	8 (50.0)	4 (25.0)
Neutropenic fever	2 (8.7)	0 (0)	4 (19.0)	0 (0)	1 (6.25)	0 (0)
Thrombocytopenia	0 (0)	0 (0)	3 (14.3)	1 (4.2)	0 (0)	0 (0)
Gastrointestinal disorders						
Abdominal discomfort	0 (0)	0 (0)	1 (4.2)	0 (0)	0 (0)	0 (0)
Abdominal distension	5 (21.7)	0 (0)	0 (0)	0 (0)	1 (6.3)	0 (0)
Abdominal pain	2 (8.7)	0 (0)	0 (0)	0 (0)	0 (0)	0 (0)
Acid reflux	1 (4.3)	0 (0)	0 (0)	0 (0)	0 (0)	0 (0)
Acute hemorrhoids	0 (0)	0 (0)	1 (4.2)	0 (0)	0 (0)	0 (0)
Constipation	6 (26.0)	0 (0)	3 (14.3)	0 (0)	1 (6.3)	0 (0)
Diarrhea	1 (4.3)	0 (0)	0 (0)	0 (0)	0 (0)	0 (0)
Gastralgia	0 (0)	0 (0)	2 (9.5)	0 (0)	0 (0)	0 (0)
Inappetence	0 (0)	0 (0)	1 (4.8)	0 (0)	1 (6.3)	0 (0)
Jaw pain	1 (4.3)	0 (0)	0 (0)	0 (0)	0 (0)	0 (0)
Mouth ulcer	0 (0)	0 (0)	1 (4.8)	0 (0)	0 (0)	0 (0)
Nausea	1 (4.3)	0 (0)	0 (0)	0 (0)	0 (0)	0 (0)
Stomach discomfort	1 (4.3)	0 (0)	3 (14.3)	0 (0)	0 (0)	0 (0)
Toothache	0 (0)	0 (0)	1 (4.8)	0 (0)	1 (6.3)	0 (0)
Vomiting	1 (4.3)	0 (0)	0 (0)	0 (0)	0 (0)	0 (0)
Liver injury						
Elevations in ALT	1 (4.3)	0 (0)	0 (0)	0 (0)	2 (12.5)	0 (0)
Elevations in AST	1 (4.3)	0 (0)	0 (0)	0 (0)	0 (0)	0 (0)
Psychiatric disorders						
Insomnia	1 (4.3)	0 (0)	0 (0)	0 (0)	0 (0)	0 (0)
Respiratory, thoracic, and mediastinal disorders						
Chest pain	0 (0)	0 (0)	1 (4.2)	0 (0)	1 (6.3)	0 (0)
Pharyngalgia	0 (0)	0 (0)	1 (4.2)	0 (0)	0 (0)	0 (0)

TEAE, treatment-emergent adverse event; ALT, alanine transaminase; AST, aspartate aminotransferase. Each adverse event is reported once, at the maximum grade, for each patient.

In total, 19 participants (82.6%) occurred TEAEs following Navelbine administration, and 17 individuals (80.9%) occurred TEAEs after administration of ANX in treatment cycle 1. The most common hematological toxicity was neutropenia, which was grades 3–4 in 40% of patients. Non-hematological toxicities including constipation, abdominal distension, nausea, vomiting, and diarrhea were generally mild to moderate. In this study, infusion site phlebitis was only observed in two patients (8.7%) following Navelbine administration. The schedule of vinorelbine Ton days 1 and 8 every 3 weeks employed in this study offered a similar safety profile as reported by other with i.v. vinorelbine.

### Responses

While this study was not powered for evaluation of anti-cancer efficacy, among the 24 recruited patients, 16 (66.7%) patients had their best objective response rate evaluated at the end of cycle 2 treatment. Fourteen patients (14/16, 87.5%) had stable disease, 1 patient (6.25%) had partial response, while 1 patient (6.25%) progressed.

Ten subjects completed four cycles of chemotherapy, 8 patients (80%) had stable disease, 1 patient (10%) had partial response, while 1 patient (6.25%) progressed.

## Discussion

This study has shown that the test product (ANX) is bioequivalent to the reference product (Navelbine), in terms of AUC_last_, AUC_inf_, and C_max_. The dose of vinorelbine administered in this study is within the range of vinorelbine doses that are used in routine clinical practice. To our knowledge, this is the first published study to characterize the tolerability and pharmacokinetic profile of vinorelbine in Chinese advanced NSCLC patients receiving a dose of 30 mg/m^2^ by 10 min i.v. When dose adjusted to be comparably equivalence, the pharmacokinetic parameters described here were similar to those observed after i.v. infusion (Marquet et al., 1992; Wargin and Lucas, 1994; Robieux et al., 1996; Marty et al., 2001; Khayat et al., 2004; Lush et al., 2005; Requejo et al., 2008). However, the C_max_ and AUC values in our study presented here (as shown in **Table 3**) were higher than the values previously reported for Chinese (Qian et al., 2011) and Western cancer patients (Schilling et al., 1996) (in 12 Chinese cancer patients receiving a dose of 25 mg/m^2^ by 20-min i.v. infusion, C_max _599.5 ± 243.6 ng·h/mL, and AUC_inf_ 476.7 ± 73.2 ng·h/mL; in 13 Western patients with solid tumors following i.v. administration 35 mg/m^2^, C_max _676.6 ± 220.2 ng·h/mL, and AUC_inf_ 476.7 ± 73.2 ng·h/mL). This variation might be due to differences in infusion time, the patients’ accompanying diseases, and analytical method for measuring the plasma concentration.

As shown in **Figure 2**, human pharmacokinetics of vinorelbine can be best described by a three-compartment model. As indicated by the large apparent volume of distribution seen after the i.v. administration of 30 mg/m^2^ in NSCLC patients, vinorelbine is extensively distributed to tissues. There are considerable inter-patient differences in estimated vinorelbine volume of distribution, some of which may be due to the patients’ characteristics: body surface area, gender, and platelet count before administration (Nguyen et al., 2002). CL and T_1/2_ did not vary signiﬁcantly with dosage and route of administration, which were close to the values reported in previous studies (Gebbia and Puozzo, 2005; Caffo et al., 2013).

Neutropenia was the most common AE, and all symptoms related to the drug neurotoxicity are usually reversible and recover after treatment discontinuation. A range of gastrointestinal disturbance AEs was also seen, although these were mild. During the study period, only two patient (2/23, 8.7%) using Navelbine experienced 1–3 grade infusion-site reactions. The incidence of infusion-site reactions in our study was much lower than that reported in previous studies (29%) (Yoh et al., 2007; Schupp et al., 2008). This may due to the fact that quite a number of patients received the test or reference product through a central line in current study. Further studies for the control of local venous toxicity of the new i.v. formulation are warranted. Overall, our results are consistent with previous safety data and show no new safety signal (Marty et al., 2001; Hirsh et al., 2007; Briasoulis et al., 2013; Sutiman et al., 2016; Bilir et al., 2017; Kaburaki et al., 2017).

At present, vinorelbine is available for administration in both i.v. and oral formulations. Previous studies have proved that oral form shows similar inter-individual variability, same metabolism pattern, reproducible intra-patient blood exposure, and same pharmacokinetic–pharmacodynamic relationship (Gebbia and Puozzo, 2005; Hirsh et al., 2007). Several advantages for the i.v. administration compared with the oral one have been suggested. The patients’ compliance can be better controlled when using i.v. administration. Moreover, i.v. vinorelbine may be required for patients with difficulty swallowing or to avoid absorption variability.

This pharmacokinetic study had some limitations, which should be considered. In our study there was an imbalance in the proportion of female and male subjects, with approximately 80% being male. In addition, levels of free drug and the active metabolite 4-O-deacetylvinorelbine were also not measured in our study, which may be higher in patients with liver dysfunction (Gong et al., 2019). Therefore, caution should be exercised when extrapolating these data to patients with abnormal liver function. As vinorelbine continues to remain a viable treatment option in refractory settings and continues development as part of novel combination therapeutic strategies including immunotherapy, ongoing investigations of its safety and pharmacokinetics are warranted.

## Conclusion

This study, performed in NSCLC patients, has demonstrated that this new emulsion formulation was pharmacokinetically equivalent to Navelbine. The reported adverse events for the two formulations are similar. It is assumed that ANX would improve tolerability and compliance in patients with cancer, especially those with increased risk of vein toxicity. Further study is warranted to explore its clinical application.

## Data Availability

All datasets generated for this study are included in the manuscript and the supplementary files.

## Ethics Statement

The study protocol and its amendments were reviewed and approved by the ethics committee of the First Affiliated Hospital, School of Medicine, Zhejiang University (approval no. 2012-EC-60). The trial was designed according to the current revised Declaration of Helsinki and conducted in accordance with Good Clinical Practice guidelines. Written informed consent was obtained from each participating patient before entry into the study.

## Author Contributions

All of the authors were involved in the design of the study, acquisition, analysis, or interpretation of data for the work, drafting the manuscript or revising it critically for important intellectual content. The authors were fully involved in all content and editorial decisions, were involved in all stages of manuscript development, and approved the final version.

## Conflict of Interest Statement

The authors declare that the research was conducted in the absence of any commercial or financial relationships that could be construed as a potential conflict of interest.
